# A multi-model based on radiogenomics and deep learning techniques associated with histological grade and survival in clear cell renal cell carcinoma

**DOI:** 10.1186/s13244-023-01557-9

**Published:** 2023-11-27

**Authors:** Shihui Wang, Chao Zhu, Yidong Jin, Hongqing Yu, Lili Wu, Aijuan Zhang, Beibei Wang, Jian Zhai

**Affiliations:** 1 Department of Radiology, The First Affiliated Hospital of Wannan Medical College, Wuhu, People’s Republic of China; 2 School of Health Science and Engineering, University of Shanghai for Science and Technology, Shanghai, People’s Republic of China

**Keywords:** Renal cell carcinoma, Computed tomography, Radiomics, Deep learning

## Abstract

**Objectives:**

This study aims to evaluate the efficacy of multi-model incorporated by radiomics, deep learning, and transcriptomics features for predicting pathological grade and survival in patients with clear cell renal cell carcinoma (ccRCC).

**Methods:**

In this study, data were collected from 177 ccRCC patients, including radiomics features, deep learning (DL) features, and RNA sequencing data. Diagnostic models were then created using these data through least absolute shrinkage and selection operator (LASSO) analysis. Additionally, a multi-model was developed by combining radiomics, DL, and transcriptomics features. The prognostic performance of the multi-model was evaluated based on progression-free survival (PFS) and overall survival (OS) outcomes, assessed using Harrell’s concordance index (C-index). Furthermore, we conducted an analysis to investigate the relationship between the multi-model and immune cell infiltration.

**Results:**

The multi-model demonstrated favorable performance in discriminating pathological grade, with area under the ROC curve (AUC) values of 0.946 (95% CI: 0.912–0.980) and 0.864 (95% CI: 0.734–0.994) in the training and testing cohorts, respectively. Additionally, it exhibited statistically significant prognostic performance for predicting PFS and OS. Furthermore, the high-grade group displayed a higher abundance of immune cells compared to the low-grade group.

**Conclusions:**

The multi-model incorporated radiomics, DL, and transcriptomics features demonstrated promising performance in predicting pathological grade and prognosis in patients with ccRCC.

**Critical relevance statement:**

We developed a multi-model to predict the grade and survival in clear cell renal cell carcinoma and explored the molecular biological significance of the multi-model of different histological grades.

**Key points:**

1. The multi-model achieved an AUC of 0.864 for assessing pathological grade.

2. The multi-model exhibited an association with survival in ccRCC patients.

3. The high-grade group demonstrated a greater abundance of immune cells.

**Graphical Abstract:**

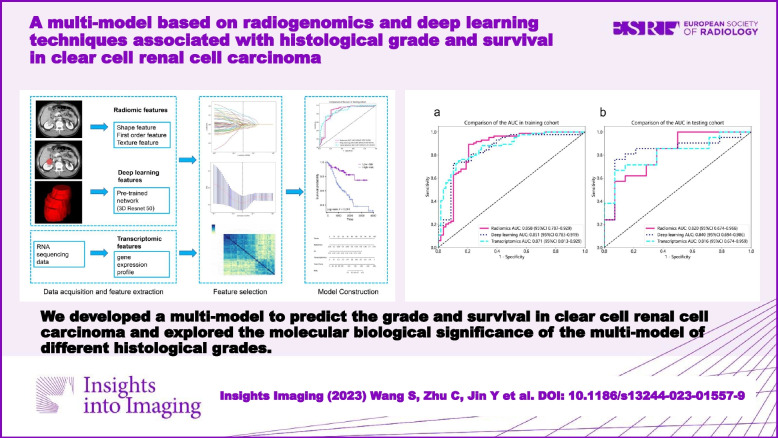

**Supplementary Information:**

The online version contains supplementary material available at 10.1186/s13244-023-01557-9.

## Introduction

Renal cell carcinoma (RCC) is a highly prevalent form of cancer globally, representing one of the most frequently diagnosed malignancies. Clear cell RCC (ccRCC) accounts for approximately 70–80% of all RCC cases [1]. The pathological grade of tumors is a critical prognostic factor for patients diagnosed with ccRCC [2, 3] and is considered a significant predictor, particularly for ccRCC. It has gained widespread recognition and is increasingly utilized to inform clinical management approaches [4, 5]. Therefore, discriminating ccRCC grade is important for personalized precision medicine. Although pathology is the gold standard for grading ccRCC [6], the percutaneous biopsy is the commonly employed technique for preoperative prediction of ccRCC grade. Nevertheless, this procedure is vulnerable to potential errors arising from sampling limitations and inter-observer variability [7, 8]. Also, inaccuracies in grade can result from sample error and tumor heterogeneity [9].

In recent years, computed tomography (CT) is the most commonly used imaging technique for examining kidney cancer due to its accuracy in both detecting and diagnosing kidney masses. Multiphase contrast-enhanced CT examination is convenient and has superior resolution, which permits clear visualization of lesions [10]. Radiomics and deep learning (DL) techniques have been increasingly utilized to predict the grade of ccRCC [11–13]. However, few studies have integrated different predictors from diverse dimensions, such as transcriptomics, which could provide valuable information for enhanced risk assessment. Transcriptomics plays a critical role in cancer diagnosis and treatment [14]. To date, no investigation has merged radiomics, DL techniques, and transcriptomics to determine ccRCC grade. Therefore, the objective of this study was to develop a multi-model that integrates radiomics, DL, and transcriptomics features to predict the grade and survival of patients with ccRCC. Furthermore, we explored the molecular biological significance of the multi-model and the immune cell infiltration in patients of different histological grade.

## Methods

### Patients and study design

The ethics committee of the hospital granted approval for this retrospective study, and the need for written informed consent was waived. The Cancer Genome Atlas Kidney Clear Cell Carcinoma dataset comprising 237 ccRCC patients was obtained from The Cancer Imaging Archive (TCIA) [15, 16]. Patient characteristics, including age, gender, pathological grade, tumor-node-metastasis (TNM) stage, and follow-up data, were obtained from TCIA. Histological grade was classified as low (grades 1–2) and high (grades 3–4) [12, 17].

**Fig. 1:**
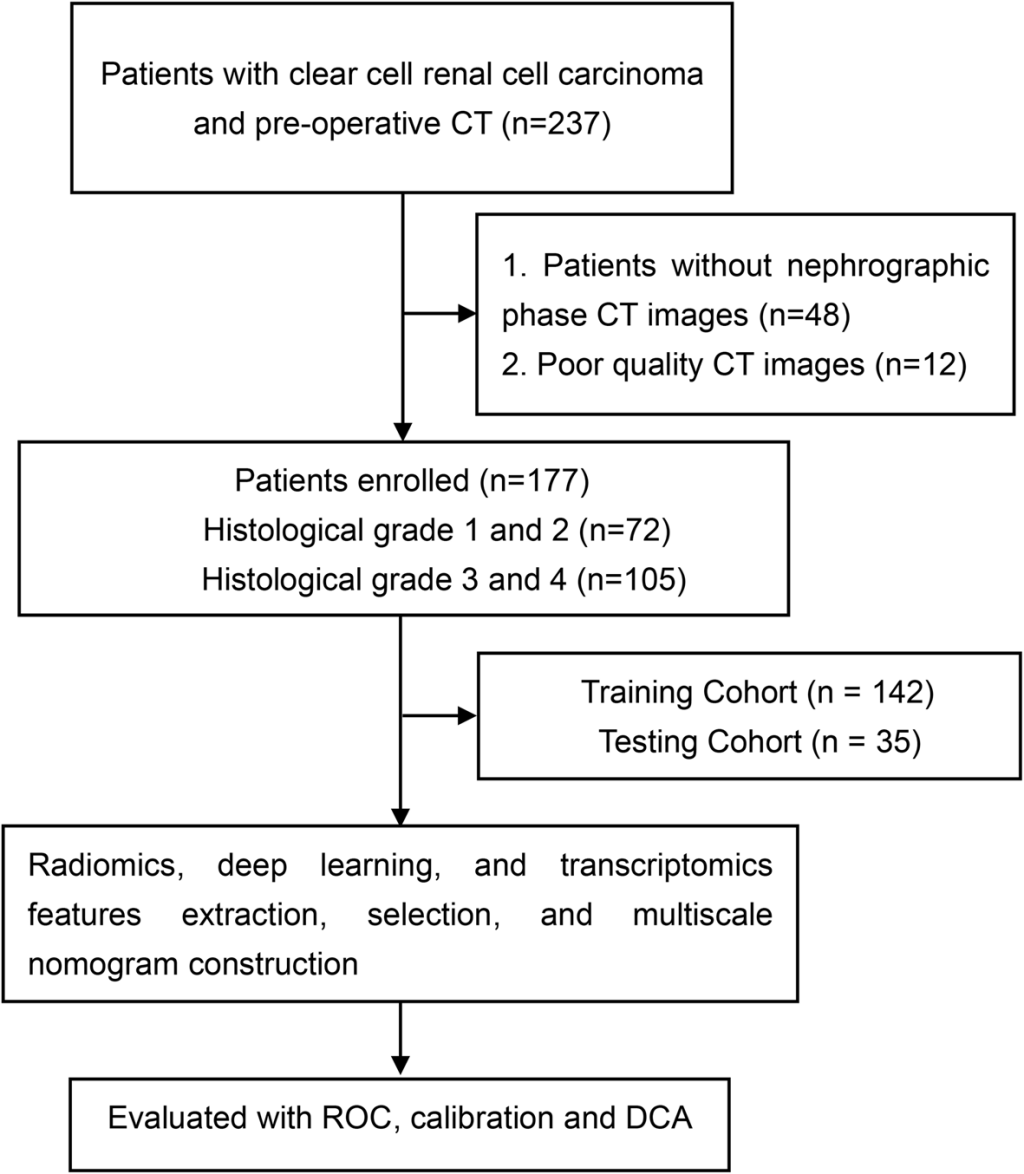
The flow diagram of the study

Figure 1 illustrates the recruitment pathway for patients in this study. A total of 177 patients with ccRCC were included, with 142 patients in the training group and 35 patients in the testing group at a randomization ratio of 8:2. Inclusion criteria consisted of the following: (1) patients diagnosed with ccRCC, (2) patients who underwent CT-enhanced scans, and (3) availability of complete genetic and clinical information. Exclusion criteria included the following: (1) patients without nephrographic phase CT images and (2) poor-quality CT images.

### Segmentation and the extraction of radiomics features

The nephrographic phase CT images were employed for radiomics feature extraction [12]. Layer-by-layer delineation of the volume of interest (VOI) was performed using ITK-SNAP software (version 3.8, www.itksnap.org/) by two radiologists, each having over 5 years of experience in diagnostic abdominal imaging. The radiologists were blinded to the patients’ pathological grade. A total of 1834 radiomics features were extracted in Python (version 3.6.0) using PyRadiomics (version 3.0.1) from the VOI for each patient with ccRCC. The reliability of the radiomics features was assessed by calculating inter- and intra-class correlation coefficients (ICCs). Radiomics features with ICCs > 0.75 were deemed reliable. For additional details on the ICC analysis, please refer to the Supplementary Material.

### DL feature extraction

In this study, a three-dimensional (3D) DL model using the 3D ResNet50 architecture was employed. The VOI was selected as the original image and resized to 96 × 96 × 96 to align with the network’s input size. The model training process consisted of updating the network weights using a cross-entropy loss function, which was utilized for the prediction task. The 3D DL model was then used to extract DL features from each VOI. For each patient in the training and testing groups, a total of 1024 DL features were extracted from the penultimate fully connected layer. All were run in Python (version 3.6.8). We used the PyTorch framework to train the model on NVIDIA RTX 3070 Ti graphics processing units. The network optimization was performed using the Adam optimizer with a learning rate of 0.001. The training process spanned 300 epochs, with a batch size of 4.

### Functional enrichment analysis

Transcriptomic data from 142 ccRCC patients were obtained from the TCGA database for genetic analysis. The differential expression of genes (DEGs) between high-grade and low-grade ccRCC samples was analyzed using the “DEseq2” package in R software. Subsequently, a Gene Ontology (GO) enrichment analysis was conducted on the DEGs to identify biological processes, cellular components, and molecular functions that exhibited significant enrichment in one group compared to the other.

### Radiomics, DL, and transcriptomics feature selection and models building

The analysis proceeded in three main steps. First, univariate regression analysis was employed to identify the radiomics, DL, and transcriptomics features that were significantly associated with grade and prognosis. Second, the least absolute shrinkage and selection operator (LASSO) method was applied to the training group in order to select the most important features. Finally, the selected important features were utilized to construct the radiomics, DL, and transcriptomics models.

### Performance of the three models and multi-model

**Fig. 2:**
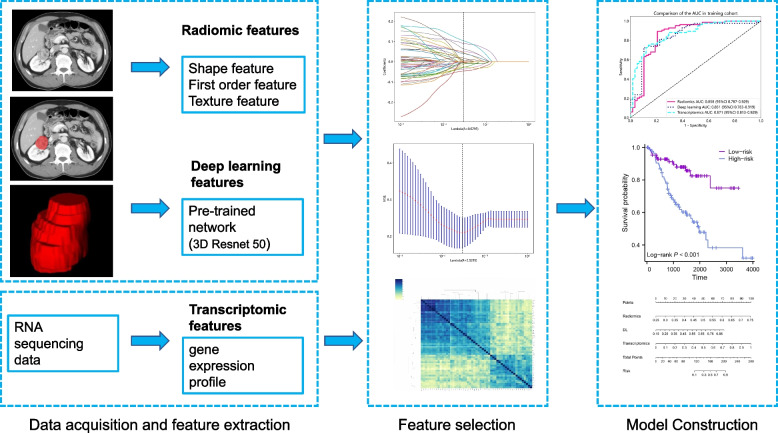
The workflow of the basic steps in multi-model development

Figure 2 illustrates the workflow encompassing the fundamental steps in radiomics development. A multi-model was created by integrating radiomics, DL, and transcriptomics models through logistic regression. To evaluate the performance of these models, metrics such as the area under the receiver operating characteristic (ROC) curve (AUC), calibration curve, and decision curve analysis (DCA) were utilized for both the training and testing datasets.

### Survival analysis and immune cells infiltration analysis

Patients were initially stratified into high-risk or low-risk groups using the median scores obtained from the multi-model. Subsequently, follow-up data was analyzed to determine progression-free survival (PFS) and overall survival (OS) outcomes. PFS was defined based on the occurrence of new tumor events, including disease progression, local recurrence, distant metastasis, or death, while OS was calculated from the date of disease diagnosis until either death or the specified cut-off date for follow-up. To visually represent the survival status of the high-risk and low-risk patient groups, Kaplan–Meier plots were generated. The prognostic potential of the multi-model and the survival status of the patients were evaluated using Harrell’s concordance index (C-index).

Enrichment scores for specific immune cells in ccRCC were calculated using Single Sample Gene Set Enrichment Analysis (ssGSEA) in R software for each patient. Additionally, a comparison of the enrichment scores of immune cell infiltration was performed between high-risk and low-risk patients. This analysis aimed to examine the association between the multi-model and histological grade, shedding light on the relationship between the predictive model and immune cell composition.

### Statistical analysis

Continuous variables that exhibited a normal distribution were reported as mean and standard deviation. Categorical variables were compared using chi-square tests, while independent samples *t*-test or Mann–Whitney *U* test was utilized to compare continuous variables. Statistical significance was considered when the *p* value was less than 0.05. The statistical analyses were conducted using Python (version 3.6.8) and R software (version 4.2.2).

## Results

### Clinical characteristics

In this study, 177 patients were diagnosed with ccRCC, with 72 having low-grade and 105 having high-grade tumors. There were 22 patients with metastasis (M-stage) and 23 patients with lymph node metastasis (N-stage) above 0. Table 1 provides an overview of the clinical characteristics of these two groups, including the training and testing cohorts. The analysis revealed no statistically significant differences in patient age and gender between the low-grade and high-grade groups (*p* > 0.05). However, there was a significant difference in the TNM stage, with the low-grade group demonstrating a lower TNM stage compared to the high-grade group (*p* < 0.05).

**Table 1:** Clinical factors of in the training and testing cohorts

Clinical factors	Training cohort (*n* = 142)			Testing cohort (*n* = 35)		
	Low-grade	High-grade	*p* value	Low-grade	High-grade	*p* value
Age (years), mean ± SD	60.345 ± 12.786	61 ± 12.502	0.761	56.571 ± 11.175	59 ± 10.578	0.520
Gender, *n* (%)			0.201			0.737
Female	24 (16.9%)	26 (18.3%)		5 (14.3%)	9 (25.7%)	
Male	34 (23.9%)	58 (40.8%)		9 (25.7%)	12 (34.3%)	
TNM stage *n* (%)			< 0.001			0.016
1–2	47 (33.1%)	39 (27.5%)		12 (34.3%)	9 (25.7%)	
3–4	11 (7.7%)	45 (31.7%)		2 (5.7%)	12 (34.3%)	

*Abbreviations*: *TNM* tumor-node-metastasis, *SD* standard deviation

### Construction of radiomics, DL, and transcriptomics models

For each patient, 1834 radiomics features were extracted from the ROIs on the CT images. After conducting univariate logistic analysis, 398 radiomics features exhibited statistically significant differences between the low-grade and high-grade groups. These features were further subjected to LASSO, which identified the 17 most valuable features (Supplementary Table 1). Based on these most valuable features and genes, three models were established using tenfold cross-validation. The radiomics model achieved AUCs of 0.858 (95% confidence interval [CI]: 0.787–0.929) and 0.820 (95% CI: 0.674–0.966) in the training and testing cohorts, respectively (Table 2).

**Table 2:** Performance of the radiomics model, deep learning model, and transcriptomics model in the training and testing cohorts

Different models	Training cohort (*n* = 142)				Testing cohort (*n* = 35)			
	AUC (95%CI)	SEN	SPE	ACC	AUC (95%CI)	SEN	SPE	ACC
Radiomics model	0.858 (0.787–0.929)	0.964	0.569	0.803	0.820 (0.674–0.966)	0.752	0.500	0.771
Deep learning model	0.851 (0.783–0.919)	0.726	0.897	0.796	0.840 (0.694–0.986)	0.762	0.929	0.829
Transcriptomics model	0.871 (0.813–0.929)	0.750	0.862	0.800	0.816 (0.674–0.959)	0.667	0.929	0.771
Combined model	0.946 (0.912–0.980)	0.952	0.862	0.916	0.864 (0.734–0.994)	0.857	0.643	0.771

*Abbreviations*: *AUC* area under the curve, *SEN* sensitivity, *SPE* specificity, *ACC* accuracy, *95% CI* 95% confidence interval

**Fig. 3:**
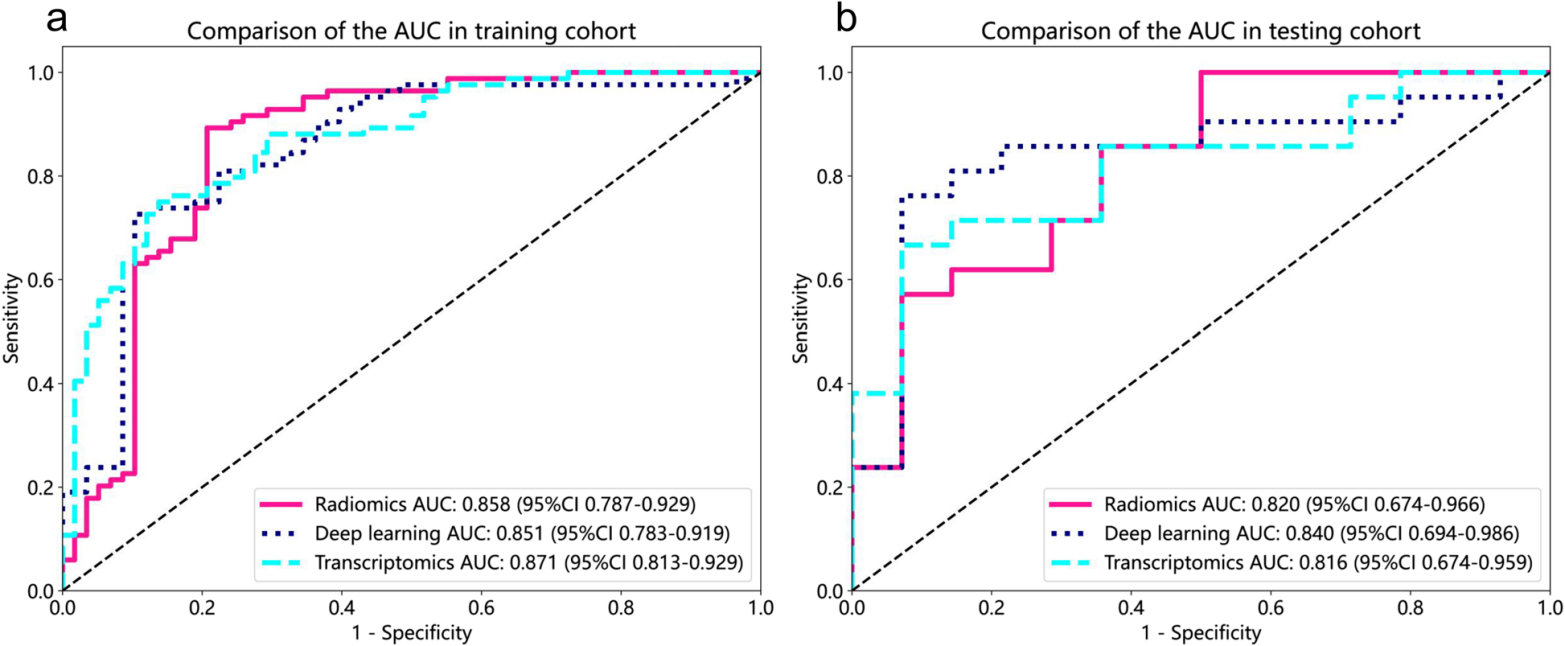
The receiver operating characteristic (ROC) curves of radiomics, deep learning, and transcriptomics models in the study cohorts. **a** ROC curves in training cohort. **b** ROC curves in testing cohort

Similarly, we obtained 9 DL features and 16 genes (Supplementary Tables 2 and 3) and established the DL and transcriptomics models. The performance of these two models is presented in Table 2. The ROC curves of the three models in the training cohort and testing cohort are shown in Fig. 3a, b.

### Performance and biologic function of the multi-model

**Fig. 4:**
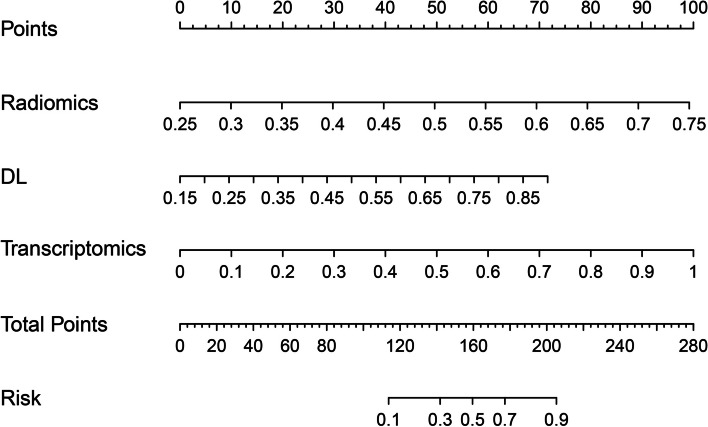
Study multi-model for outcome prediction

In this study, we developed a multi-model that integrated radiomics, DL, and transcriptomics models. The model exhibited strong predictive performance, with AUCs of 0.946 (95% CI: 0.912–0.980) and 0.864 (95% CI: 0.734–0.994) in the training and testing cohorts, respectively (Table 2). Figure 4 illustrates the constructed multi-model. The calibration curve of the model demonstrated satisfactory calibration, indicating good agreement between predicted and observed outcomes (Supplementary Fig. 1a). Furthermore, the DCA curves revealed that the model improved the ability to distinguish between low-grade and high-grade tumors (Supplementary Fig. 1b).

**Fig. 5:**
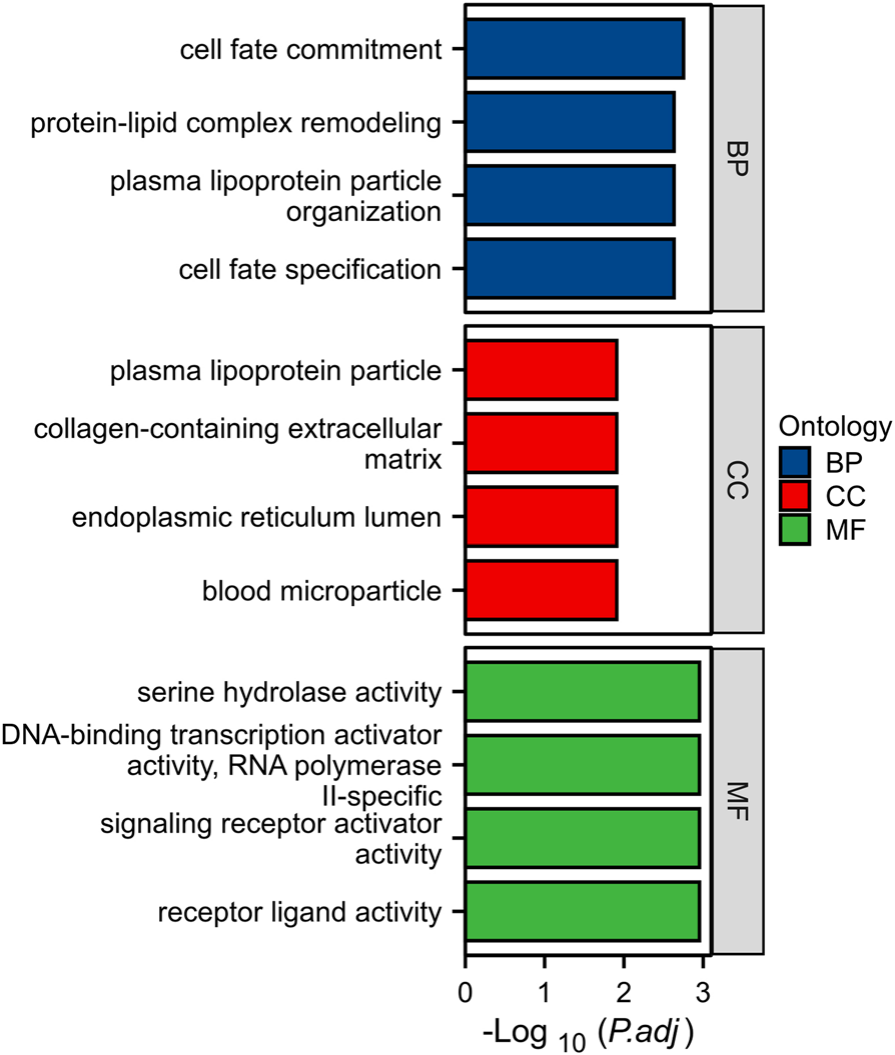
Gene ontology enrichment analysis of differentially expressed genes in the low-grade and high-grade group

RNA sequencing analysis and functional enrichment analysis showed significant differences in transcriptional aspects between high-grade and low-grade in patients with ccRCC (Fig. 5).

### Prognostic value and immune cell infiltration of multi-model

**Fig. 6:**
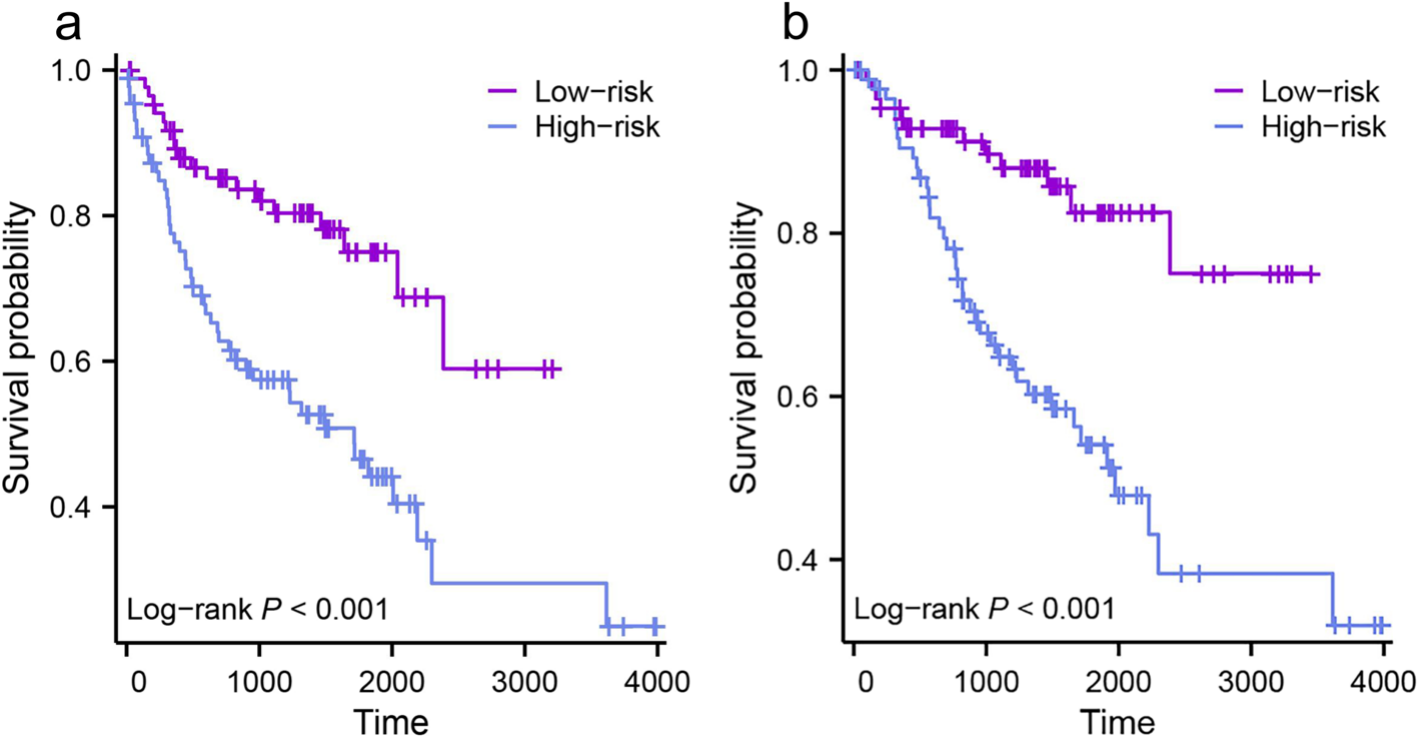
Kaplan–Meier (KM) plots of the survival status of the high-risk and low-risk patient groups. **a** KM curves for progression-free survival. **b** KM curves for overall survival

Kaplan–Meier analysis demonstrated that patients with ccRCC and high-risk model-scores experienced a significantly shorter survival time in terms of PFI compared to those with low-risk model-scores (Fig. 6a). Additionally, patients with high-risk model-scores exhibited poorer OS time compared to those with low-risk model-scores (Fig. 6b). We also constructed a clinical prediction model using pathological low/high grade and TNM stage. The prognostic value of our multi-model scores and clinical model was assessed based on follow-up data. The predictive performance of our multi-model exhibited a slight inferiority compared to that of the clinical model in both the PFS (C-index = 0.62 vs. 0.71) and the OS (C-index = 0.63 vs. 0.68). A combined model was further formulated by integrating the multi-model with the clinical model. The combined model achieved high predictive accuracy for PFS (C-index = 0.74; 95% CI, 0.71 to 0.77) and OS (C-index = 0.72; 95% CI, 0.69 to 0.75).

In addition to analyzing the relationship between the multi-model and histological grade, we also compared the differences in immune cell subtypes between the high-risk and low-risk groups. The analysis revealed that immune cell abundance was greater in the high-risk group compared to the low-risk group (Supplementary Fig. 2). Specifically, the high-risk group exhibited higher enrichment scores for activated CD4 T cells (*p* = 0.005), activated CD8 T cells (*p* = 0.002), activated dendritic cells (*p* < 0.001), and central memory CD4 T cells (*p* < 0.001) (Supplementary Fig. 3).

## Discussion

In this study, we developed a multi-model for preoperative identification of the histological grade of ccRCC. The multi-model contains radiomics features, DL features, and transcriptomics features. We then further investigated the immune cell infiltration and prognostic value of the multi-model. Our study showed that this CT-based multi-model achieved favorable performance in predicting the histological grade and survival of ccRCC.

The prognosis for a high-grade ccRCC is poor and a clear histological grade is essential to monitor the patient’s condition and to develop an individualized follow-up treatment strategy [18–20]. With the development of radiomics and DL techniques, studies have focused on images for preoperative non-invasive prediction of the histological grade of ccRCC [21–23]. Demirjian et al. [12] developed a CT-based radiomics model to discriminate between high-grade and low-grade and showed that a classification model based on 10 radiomics features achieved an AUC of 0.73. Zheng et al. [17] developed and validated a novel CT-based model method for preoperative prediction of ccRCC grade by combining radiomics features and CT-determined T-staging. The model method offers a non-invasive and convenient tool that promises to be an efficient aid to clinical decision-making for patients with ccRCC. These two studies mentioned above provide good research ideas in terms of radiomics to predict the histological grade. However, more computer technology is being used by academics. An effective, time-saving DL method incorporating self-supervised learning has been constructed to identify patients with high-grade [21]. Furthermore, we constructed a multi-model combined by radiomics, DL, and transcriptomics features to predict the histological grade of ccRCC.

The primary objective of our study was to develop a CT-based multi-model capable of predicting the histological grade of ccRCC. The multi-model achieved high predictive performance, as evidenced by AUCs of 0.946 and 0.864 in the training and test sets, which demonstrated promising performance in predicting grades. We also found that the high and low scores derived from the multi-model patients’ PFI and OS (*p* < 0.001) suggest that our model can predict patient prognosis [24, 25]. Secondly, we explored the molecular biological significance of the multi-model and the immune cell infiltration in patients of different histological grades. Functional enrichment analysis showed that patients with high-grade ccRCC were more transcriptionally active [26]. Additionally, our analysis demonstrated a significant increase in immune cell infiltration within the tumor microenvironment among patients classified as high-risk, compared to those in the low-risk group. This finding suggests that immune cell infiltration might play a relevant role in disease development and progression [27, 28].

This study has several limitations that need to be acknowledged. Firstly, the sample size in our study was relatively small. Secondly, this is a retrospective design of the study. Therefore, further validation of our results is necessary through prospective multicenter studies with larger sample sizes to improve the developed multi-model.

## Conclusions

Our study introduced an innovative amalgamation of radiomics, deep learning, and transcriptomics features, demonstrating the potential to predict pathological grade and prognosis in ccRCC patients. Conducting prospective multicenter studies in the future to validate our findings could offer increased confidence in patient management.

### Supplementary Information

**Additional file 1:** **Supplementary Table 1. **Radiomics features selected in the training cohort. **Supplementary Table 2.** transcriptomics features selected in the training cohort. **Supplementary Table 3.** Deep learning features selected in the training cohort. **Supplementary Fig. 1a.** The calibration curve of multi-model in the training and testing cohorts. b The decision curve analysis of multi-model in the training and testing cohorts. **Supplementary Fig. 2.** Bar graphs of immune cell infiltration in the low-risk (group 1) and high-risk (group 2) groups in the multi-model. **Supplementary Fig. 3.** Scatter plot of immune cell infiltration in the low-risk (group 1) and high-risk (group 2) groups in the multi-model.

## Data Availability

The data underlying this article cannot be shared publicly due to the privacy of individuals that participated in the study. The data will be shared on reasonable request to the corresponding author.

## Abbreviations

3D: Three-dimensional
AUC: Area under the ROC curve
C-index: Harrell’s concordance index
ccRCC: Clear cell renal cell carcinoma
CT: Computed tomography
DCA: Decision curve analysis
DL: Deep learning
GO: Gene ontology
LASSO: Least absolute shrinkage and selection operator
OS: Overall survival
PFS: Progression-free survival
ROC: Receiver operator characteristic
ssGSEA: Single Sample Gene Set Enrichment Analysis
TCIA: The Cancer Imaging Archive
TNM: Tumor-node-metastasis
VOI: Volume of interest
