# Supplementary material for: A multi-model based on radiogenomics and deep learning techniques associated with histological grade and survival in clear cell renal cell carcinoma
Source: Insights Imaging. 2023 Nov 27;14:207. doi: 10.1186/s13244-023-01557-9 (PMC10682311; doi:10.1186/s13244-023-01557-9)

**A multi-model based on radiogenomics and deep learning techniques associated with histological grade and survival in clear cell renal cell carcinoma**

**ELECTRONIC SUPPLEMENTARY MATERIAL**

**Evaluate Inter- and intra-class correlation coefficients (ICCs)**

Thirty samples were randomly selected from the registered patients to evaluate the intra- and inter-observer agreement. To evaluate intra-observer repeatability, radiologist 1 delineated twice in two weeks. To assess inter-observer repeatability, radiologist 2 separately carried out VOI delineations once. When Inter- and intra-class correlation coefficients (ICCs) values were greater than 0.75, this indicated good agreement between the extracted features. The remaining samples segmentation was performed by radiologist 1 alone.

**Supplementary Table 1** Radiomics features selected in the training cohort

| Numbers | Features | Coefficient |
| --- | --- | --- |
| Feature_1 | exponential_glszm_SizeZoneNonUniformity | 0.023882 |
| Feature_2 | gradient_firstorder_Minimum | -0.011983 |
| Feature_3 | lbp_3D_k_firstorder_Minimum | -0.016345 |
| Feature_4 | lbp_3D_k_glcm_ClusterShade | 0.057198 |
| Feature_5 | lbp_3D_k_glszm_SmallAreaLowGrayLevelEmphasis | 0.011871 |
| Feature_6 | lbp_3D_m1_glcm_JointAverage | 0.018119 |
| Feature_7 | lbp_3D_m1_glrlm_GrayLevelNonUniformityNormalized | -0.008349 |
| Feature_8 | lbp_3D_m2_glcm_Imc2 | -0.000473 |
| Feature_9 | lbp_3D_m2_ngtdm_Complexity | 0.027152 |
| Feature_10 | original_shape_Sphericity | -0.034634 |
| Feature_11 | square_glszm_LowGrayLevelZoneEmphasis | -0.009682 |
| Feature_12 | wavelet_HLH_firstorder_Median | 0.008116 |
| Feature_13 | wavelet_HLH_firstorder_RootMeanSquared | 0.034191 |
| Feature_14 | wavelet_HLH_glcm_Idn | 0.002338 |
| Feature_15 | wavelet_HLL_glcm_ClusterProminence | 0.003340 |
| Feature_16 | wavelet_LHL_firstorder_Skewness | -0.025480 |
| Feature_17 | wavelet_LLH_glcm_Imc2 | -0.013149 |

**Supplementary Table 2** transcriptomics features selected in the training cohort

| Numbers | Features | Coefficient |
| --- | --- | --- |
| Feature_1 | UNC5D | 0.023882 |
| Feature_2 | PITX2 | -0.011983 |
| Feature_3 | EDN2 | -0.016345 |
| Feature_4 | AVPR1A | 0.057198 |
| Feature_5 | AJAP1 | 0.011871 |
| Feature_6 | NECAB2 | 0.018119 |
| Feature_7 | PYCR1 | -0.008349 |
| Feature_8 | SMLR1 | -0.000473 |
| Feature_9 | BDNF | 0.027152 |
| Feature_10 | SIX2 | -0.034634 |
| Feature_11 | TEKT2 | -0.009682 |
| Feature_12 | GDPD3 | 0.008116 |
| Feature_13 | PIK3R6 | 0.034191 |
| Feature_14 | GJA4 | 0.002338 |
| Feature_15 | PLA1A | 0.003340 |
| Feature_16 | LILRA6 | -0.025480 |

**Supplementary Table 3** Deep learning features selected in the training cohort

| Numbers | Features | | Coefficient |
| --- | --- | --- | --- |
| Feature_1 | DL_Feature_1 | 0.099125 | |
| Feature_2 | DL_Feature_2 | -0.002408 | |
| Feature_3 | DL_Feature_3 | 0.004512 | |
| Feature_4 | DL_Feature_4 | -0.024271 | |
| Feature_5 | DL_Feature_5 | 0.019543 | |
| Feature_6 | DL_Feature_6 | -0.003816 | |
| Feature_7 | DL_Feature_7 | -0.028886 | |
| Feature_8 | DL_Feature_8 | -0.031921 | |
| Feature_9 | DL_Feature_9 | -0.010182 | |

**Supplementary Fig. 1** **a** The calibration curve of multi-model in the training and testing cohorts. **b** The decision curve analysis of multi-model in the training and testing cohorts.


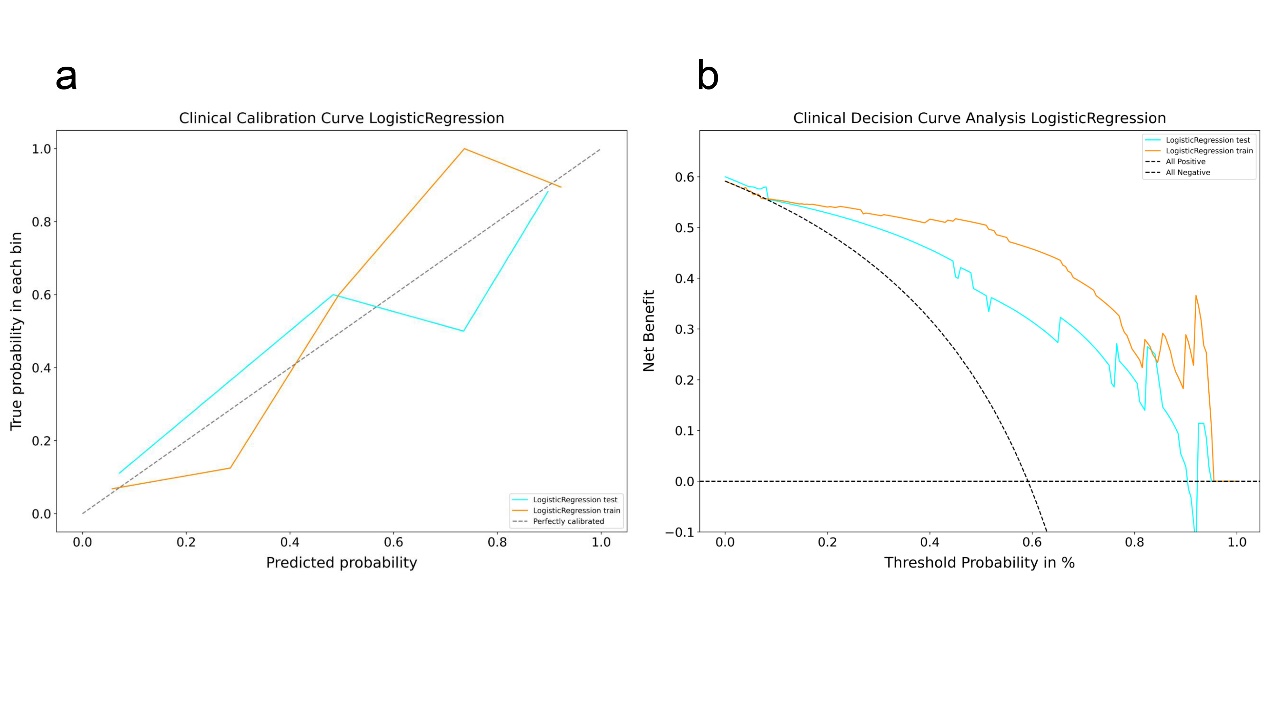


**Supplementary Fig. 2** Bar graphs of immune cell infiltration in the low-risk (group 1) and high-risk (group 2) groups in the multi-model.


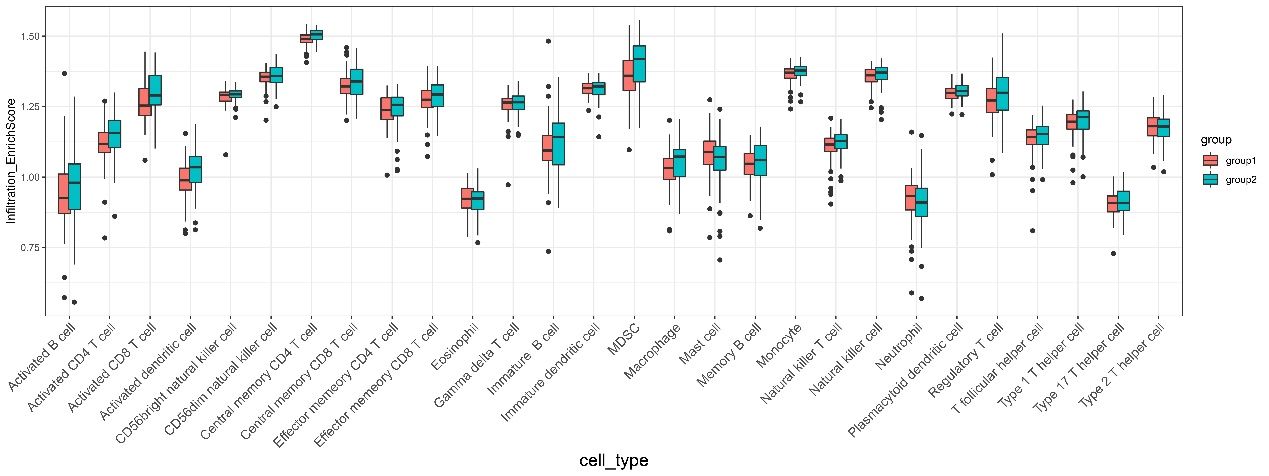


**Supplementary Fig. 3** Scatter plot of immune cell infiltration in the low-risk (group 1) and high-risk (group 2) groups in the multi-model.


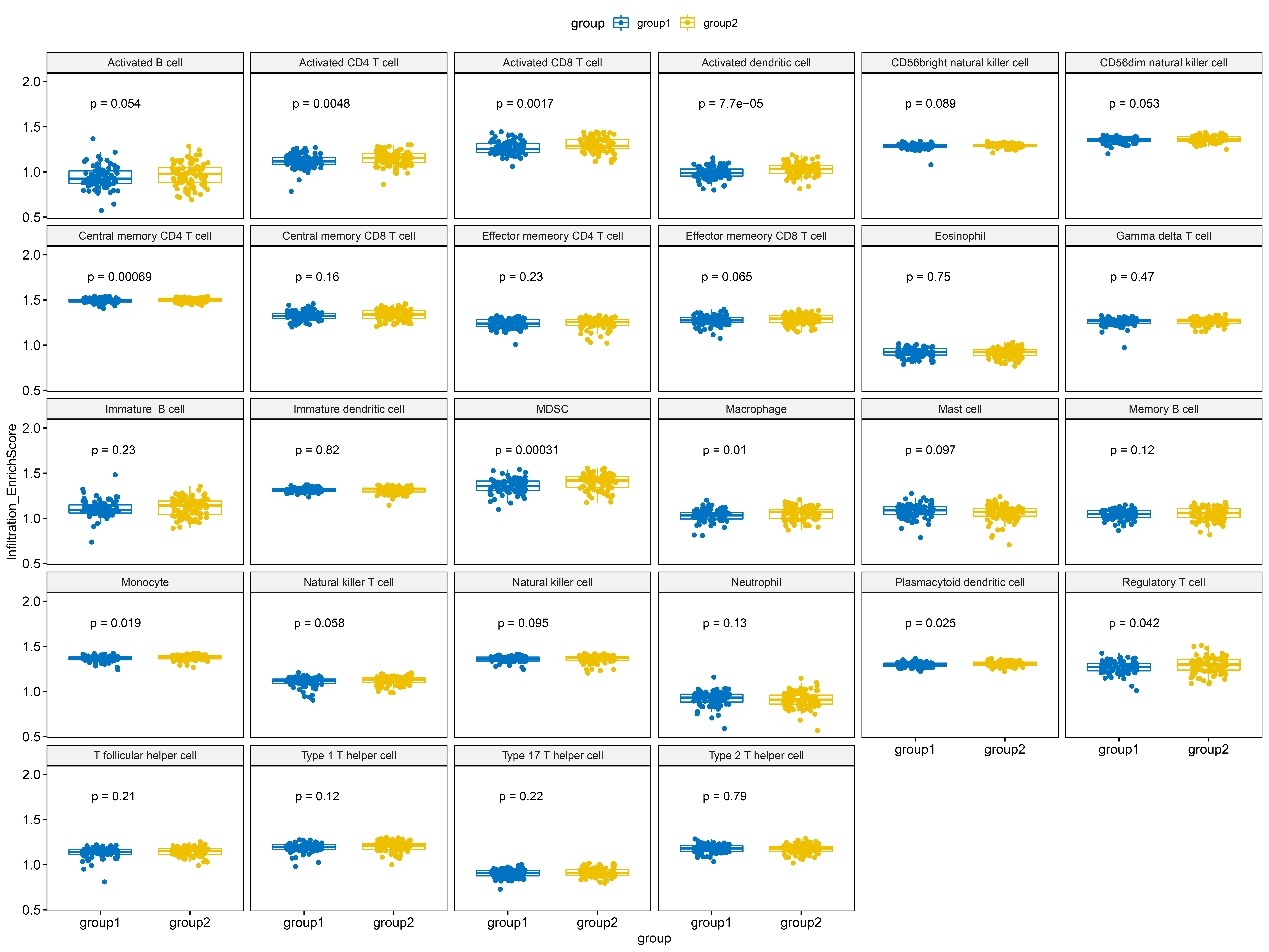

Supplement: Supplementary file 1 — Additional file 1: Supplementary Table 1. Radiomics features selected in the training cohort. Supplementary Table 2. transcriptomics features selected in the training cohort. Supplementary Table 3. Deep learning features selected in the training cohort. Supplementary Fig. 1a. The calibration curve of multi-model in the training and testing cohorts. b The decision curve analysis of multi-model in the training and testing cohorts. Supplementary Fig. 2. Bar graphs of immune cell infiltration in the low-risk (group 1) and high-risk (group 2) groups in the multi-model. Supplementary Fig. 3. Scatter plot of immune cell infiltration in the low-risk (group 1) and high-risk (group 2) groups in the multi-model. [file 13244_2023_1557_MOESM1_ESM.docx]
